# Design and Development of a Novel 3-D Printed External Fixation Device for Fracture Stabilization

**DOI:** 10.1186/s41205-023-00179-7

**Published:** 2023-06-14

**Authors:** Nathan Wm. Skelley

**Affiliations:** Sanford Health, The University of South Dakota School of Medicine Sioux Falls, 1210 West 18th Street VanDemark Building Suite G01, Sioux Falls, SD 57104 USA

**Keywords:** 3D printing, 3-dimensional printing, External fixation, Fracture, Stabilization, Orthopedics, Orthopaedics, trauma

## Abstract

**Background:**

An external fixator is an orthopaedic device used to stabilize long bone fractures after high energy trauma. These devices are external to the body and fixed to metal pins going into non-injured areas of bone. They serve a mechanical function to maintain length, prevent bending, and resist torque forces about the fracture area. The purpose of this manuscript is to describe a design and prototyping process creating a low-cost entirely 3-D printed external fixator for fracture stabilization of extremity fractures. The secondary objective of this manuscript is to facilitate future advancements, modifications, and innovations in this area of 3-D printing in medicine.

**Methods:**

This manuscript describes the computer aided design process using desktop fused deposition modeling to create a 3-D printed external fixator system designed for fracture stabilization. The device was created using the orthopaedic goals for fracture stabilization with external fixation. However, special modifications and considerations had to be accounted for given the limitations of desktop fused deposition modeling and 3-D printing with plastic polymers.

**Results:**

The presented device accomplishes the goals of creating a construct that can be attached to 5.0 mm metal pins, allows for modularity in placement orientations, and facilitates adjustable lengths for fracture care. Furthermore, the device provides length stability, prevention of bending, and resists torque forces. The device can be printed on a desktop 3-D printer using standard low-cost polylactic acid filament. The print time is less than two days and can be completed on one print bed platform.

**Conclusions:**

The presented device is a potential alternative for fracture stabilization. The concept of a desktop 3-D printed external fixator design and method of production allows for numerous diverse applications. This includes assisting areas with remote or limited access to advanced medical care and large-scale natural disasters or global conflicts where large volumes of fractures exceed the local medical supply chain capabilities. The presented device creates a foundation for future devices and innovations in this fracture care space. Further research is needed on mechanical testing and clinical outcomes with this design and initiative in fracture care before clinical application.

## Background

**Fig. 1:**
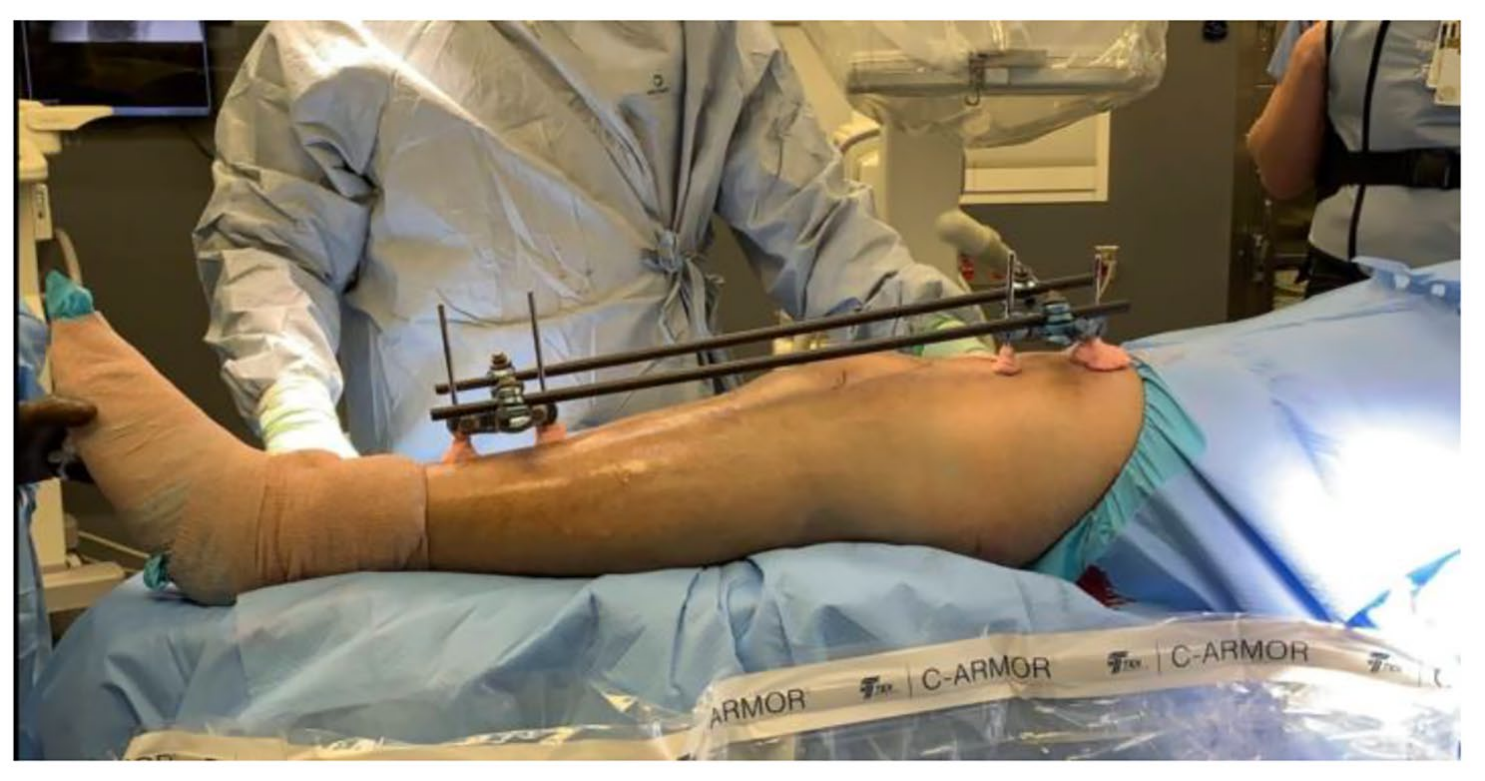
This image demonstrates a modern external fixator applied for a tibial plateau fracture around the knee. The patient has severe swelling and surgeons are unable to incise the skin because of the increased risk of soft tissue compromise and infection. Metal pins are placed in healthy bone proximal and distal to the zone of injury. The external fixator attaches to these pins with pin-to-bar clamps. These clamps secure bars that span across the zone of injury to allow swelling to subside before final fracture fixation of the tibia at a time in the future

External fixation of fractures is one of the oldest known medical procedures. From using branches and twine to immobilize extremity fractures, to the current carbon-fiber and stainless-steel reinforced devices, external fixation of broken bones has been utilized in medical procedures for more than 2000 years [1]. Although the application and technology of these devices have advanced dramatically, the mechanical goals remain the same [2]. The mechanical objective is to hold soft tissues and bone fracture fragments at stable length, prevent bending moments, and resist torsional forces about the fracture and injury zone [2] (Fig. 1).

**Fig. 2:**
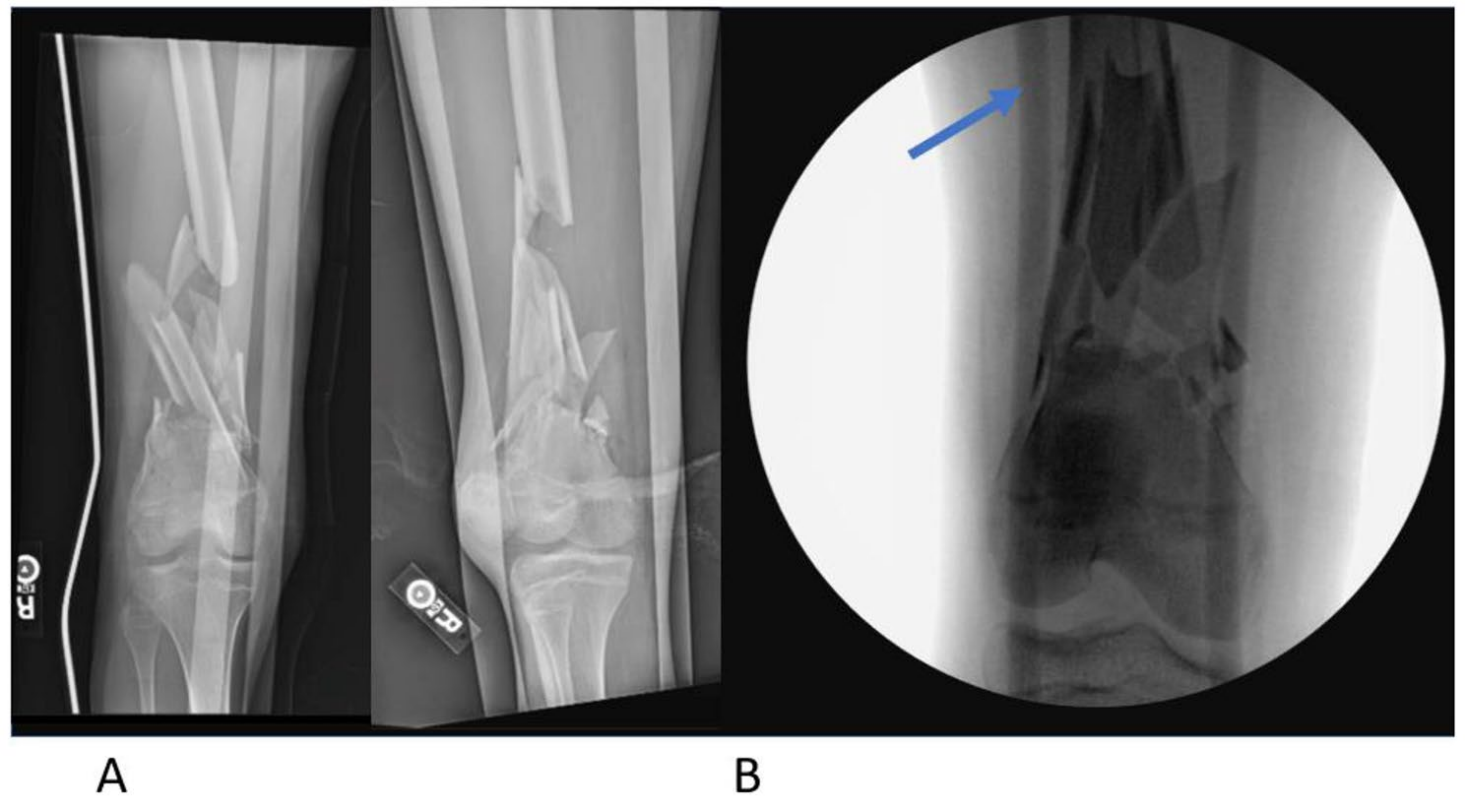
Figure **A** demonstrates an initial anterior-to-posterior and lateral x-ray of a complex femur fracture. Figure **B** demonstrates the same fracture after external fixation placement. The “blue arrow” is pointing to one of the external fixator rods spanning this area of fracture and soft tissue injury. Definitive fixation was planned two weeks later with plates and screws

After high-energy musculoskeletal trauma, there is typically severe swelling of the soft tissues around the fracture site. Orthopaedic surgeons are unable to operate on the fracture until this swelling decreases. Operating in the setting of initial trauma, with significant swelling, risks being unable to close the incisions at the conclusion of the fracture fixation surgery. This situation increases the possibility of wound complications, infections, and amputations [3]. External fixation serves the important role of spanning the fracture site, outside of the body, to stabilize the fracture and injury zone immediately after a trauma. The external fixator is an important component of damage control orthopaedics and allows the surrounding soft tissues to recover, inflammation to subside, and swelling to decrease [1, 3, 4](Fig. 2).

**Fig. 3:**
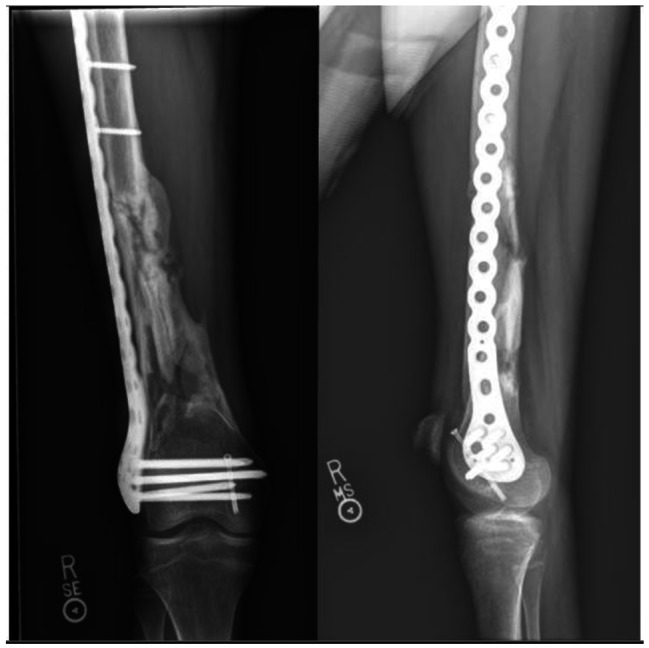
Anterior-to-posterior and lateral x-rays with definitive fixation three months after the same femur fracture demonstrated in Fig. 2. These images show fracture consolidation, healing, and the definitive fixation with plates and screws which were applied two weeks after the external fixator was initially placed at the time of trauma

External fixators can be used for definitive fixation of fractures. However, they are usually temporary, staying in place for several weeks. Once the swelling has decreased, it is an improved environment for the surgeon to definitively fix the fractures with plates, screws, intramedullary fixation, or joint replacement based on the fracture situation (Fig. 3). The external fixator is also helpful in quickly stabilizing fractures and allowing transfer to a higher level of orthopaedic care if that cannot be delivered at the same hospital where the external fixator was applied [1, 3].

When large scale natural disasters occur or global political conflicts occur, there can be many casualties. Patients that survive the initial trauma commonly have musculoskeletal injuries [5]. Large volumes of patients with fractures can quickly overwhelm hospital systems and surgical supply chains. Current industry designed external fixation devices perform the objectives of fracture stabilization very well [1, 2]. However, modern external fixators have an average cost of $5,900. The total yearly expenditure for fixator components reached $670,805 at a single level-one urban trauma center in the United State of America [6]. The costs, advanced materials, manufacturing timelines, storage demands, and limited supplies of medical grade external fixators present problems for addressing high patient volumes, reaching remote service areas without access to advanced medical care, and expenses for patients in developing low-income countries [7, 8].

Considering these problems and supply chain distribution issues, it would not be ideal to design a new low-cost external fixator based on available supplies for application in these remote and underserved populations. For example, considering an extreme situation, supplies at a local hardware store could replicate the mechanical objectives of an external fixator [2], but they are still limited in resource availability for production. Similarly, they are still limited by shipping and storage considerations for large rod-like, heavy items. Using these supplies also introduces the possibility of reproducibility issues if different supplies are utilized from different locations.

3-D printing has emerged as a unique solution for supply chain disruptions. This was especially apparent during the COVID-19 global pandemic [9–11]. 3-D printing has served numerous different applications in the field of orthopaedic surgery [12]; however, there is sparse literature or understanding of applying 3-D printing for supply chain shortages for external fixation. The 3-D printing community is collaborative and in times of large-scale disasters resulting in musculoskeletal traumas, the community could assist with scalability of device production. Similarly, as 3-D printing technology becomes much more accessible and accurate, it is easier to collaborate over files in a standardized online global environment.

Previous studies have described 3-D printing certain component parts of an external fixator [13, 14] but these works still suffer from the previously listed limitations requiring other components, such as carbon fiber rods, and shipping them to target areas. There are numerous regulatory and sterility concerns with placing a device into the body. To the author’s knowledge, there are no previous studies that have described 3-D printing the stainless steel partially threaded metal pins that fixate in the bone. These metal pins include Steinmann pins and Schanz pins or Schanz screws. These metal 5.0 mm partially threaded pins are not an expensive component of the external fixator system (~$22/each) [15] and are more commonly available in global operating theaters. They are also relatively easy to ship, store, and sterilize when needed. Thus, a novel external fixator design and system would need to interface with these metal pins. To the author’s knowledge, there are no previous reports describing 3-D printing the entire external fixator construct on a low-cost desktop 3-D printer to interface with these metal pins.

The primary purpose of this manuscript is to describe the design and prototyping process of creating a completely 3-D printed external fixator for fracture stabilization of extremity fractures. When trying to replace the current medical devices, utilizing carbon fiber and stainless steel, with readily available desktop 3-D printing filament, several re-design considerations needed to be undertaken to compensate for the weakness of plastic filament materials. Similarly, special considerations needed to be given to component design to facilitate the printing processes with Fused Deposition Modeling (FDM) desktop printers. For example, keeping scaffolding supports to a minimum to decrease post-processing work was incorporated into the design. Also, keeping all parts on two print platforms or less was important. The secondary objective of this manuscript is to facilitate future advancement, modifications, and innovations in this orthopaedic care area.

## Methods

A review was performed on current industry external fixator devices to develop design ideas and project objectives. Autodesk Fusion 360 (Autodesk Inc., San Francisco, California) was utilized to perform the computer aided design process. Each component was saved as a Standard Tessellation Language (STL) format file. The STL files were imported into PrusaSlicer Version 2.5.1 (Prusa Research, Prague, Czech Republic). Print layout was optimized to facilitate strength with the FDM printing orientation.

A goal for this project was to use 3-D printing materials and equipment that are readily available to people with basic 3-D printing experience. Once this method was established, a review was performed on common low-cost filaments and commonly utilized desktop FDM printers.

The 3-D printer selected and utilized for all prototype design and printing was the Prusa MK3S+ (Prusa Research, Prague, Czech Republic). The filament utilized in all prototypes was Overture PolyLactic Acid (PLA) 1.75 mm (Overture 3D Technologies LLC, Missouri City, Texas, USA). No modifications were made to the Prusa printer or the filament. The smooth flexible print bed was utilized for printing. Prepopulated “Prusament PLA” print settings were utilized including hot-end temperature of 215^O^C and print bed temperature of 60^O^C. Calibration for the printer was performed in industry recommended standard fashion. The prints were run at 80% speed for the first layer to facilitate adhesion and then increased to 100% speed for the remaining layers.

All file designs and 3-D printed components were assessed and inspected by the author (NWS), an American Board of Orthopaedic Surgery - Board-Certified orthopaedic surgeon in the United States of America. Inspection included visual observations, design considerations for 3-D printing, and manual loading to replicate qualities of loading experienced in the operating room when the device is applied during surgery. Devices were excluded and discarded until a device that met the following objectives was obtained:

- The device needed to attach to 5.0 mm metal pins allowing for various spacing of these pins.
- The device needed to maintain a stable length, prevent bending moments, and resist torsional forces once applied to the pins.
- The device needed to allow for various angles of application with respect to the metal pins and the device had to allow for varying lengths to accommodate a wide arrangement of fracture patterns.
- The external fixator needed to have size scalability to apply to upper and lower extremity fractures.
- To facilitate printing and limit post-processing, design iterations should focus on compact print bed layout and limit the need for scaffolding material with the prints.
- Finally, a focus should be made on 3-D printing the entire external fixator kit without needing additional supplies for application.

## Results

Medical grade trauma external fixators are comprised of three main elements; (1) Metal pins that are placed into the stable bone surrounding the fracture, (2) Clamps that attach to the metal pins, and (3) Extension rods that attach to the clamps on the pins and span the area of the fracture for stability.

The 5.0 mm stainless steel medical grade pins were maintained for this project, as previously mentioned, given that these were the only elements entering a patient’s body. The design process was then focused on creating the second and third design elements with 3-D printing and PLA.

**Fig. 4:**
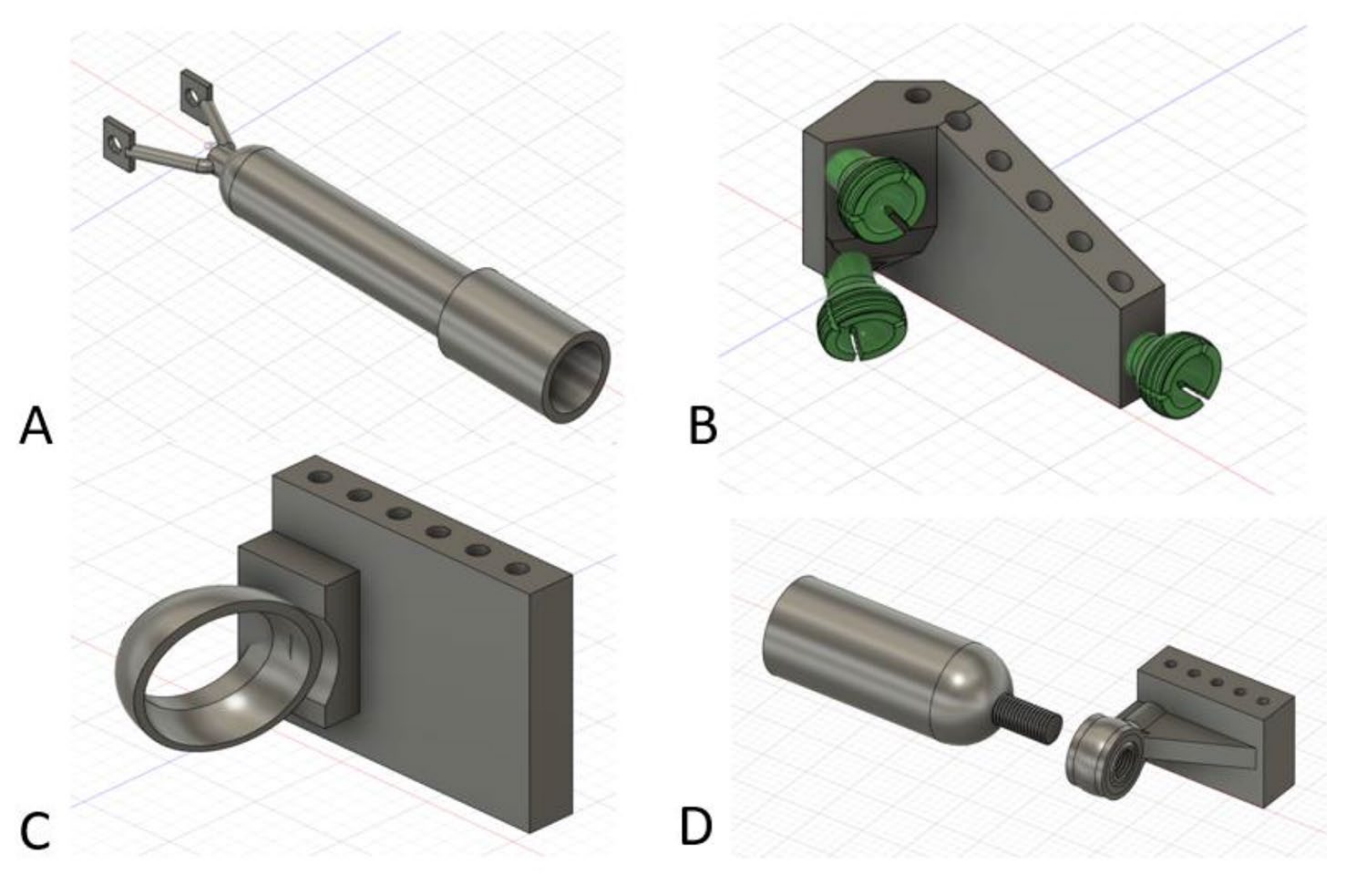
Examples of pin-to-block designs that were excluded from the final external fixator design

Figure 4 demonstrates initial design concepts creating a component to allow for attachment to the metal pins. Figure 4 A had a screw mechanism that compressed the metal pin between the “Y” tail-end. Figure 4B demonstrates multiple ball screw mechanisms that interfaced with a ball attached on an extension rod cylinder with a locking nut. Figure 4 C and 4D accommodated various spherical cylinders in cup-receiver design. In Fig. 4D, the ball had threads applied to a hole going through the floating ball inside the block assembly. This ball was free-floating within the block extension, but exhibited slippage during loading when the extension cylinder screwed into this articulating ball and was manually loaded. These Fig. 4 designs all failed with simple manual loading of the PLA prints around these attachment points. The development of modularity in this proximal pin location created a weak point in the 3-D printed PLA designs.

**Fig. 5:**
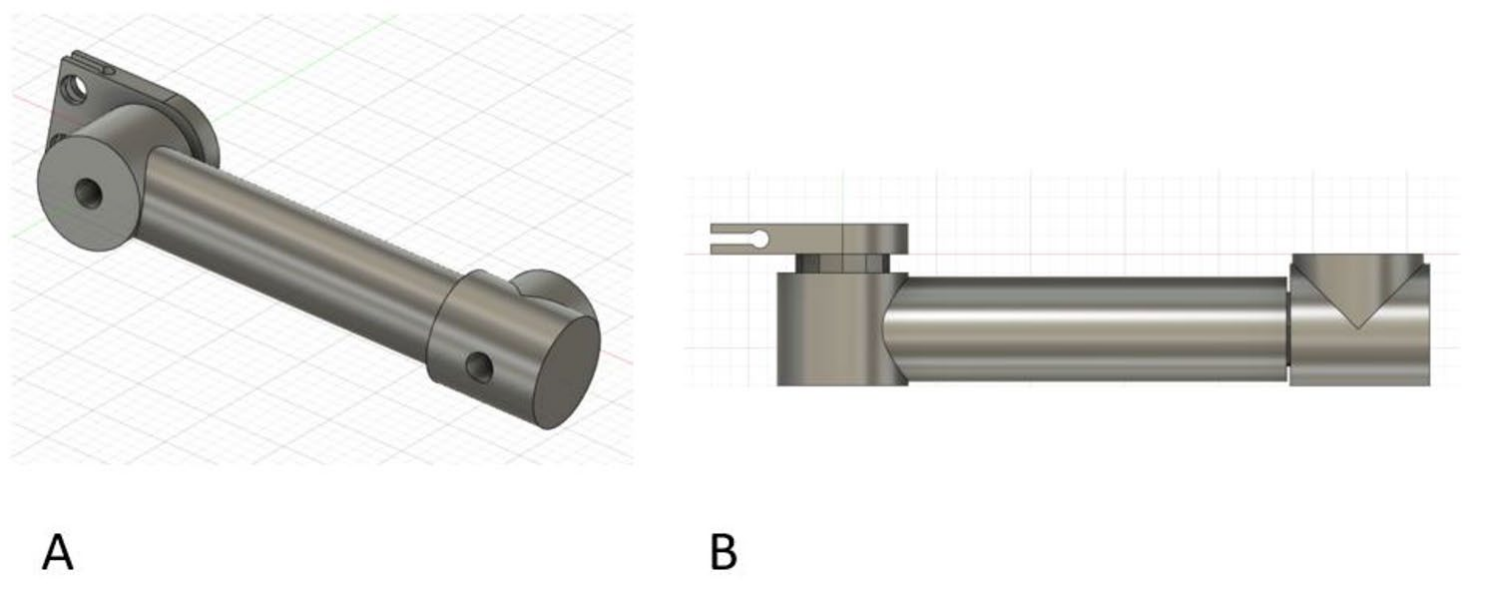
This figure demonstrates an excluded design that involved two coaxial cylinders that are fixed against a clip around the 5.0 mm metal pin. The design has several limitations, but the compression clip feature did prevent migration up or down the metal pin

**Fig. 6:**
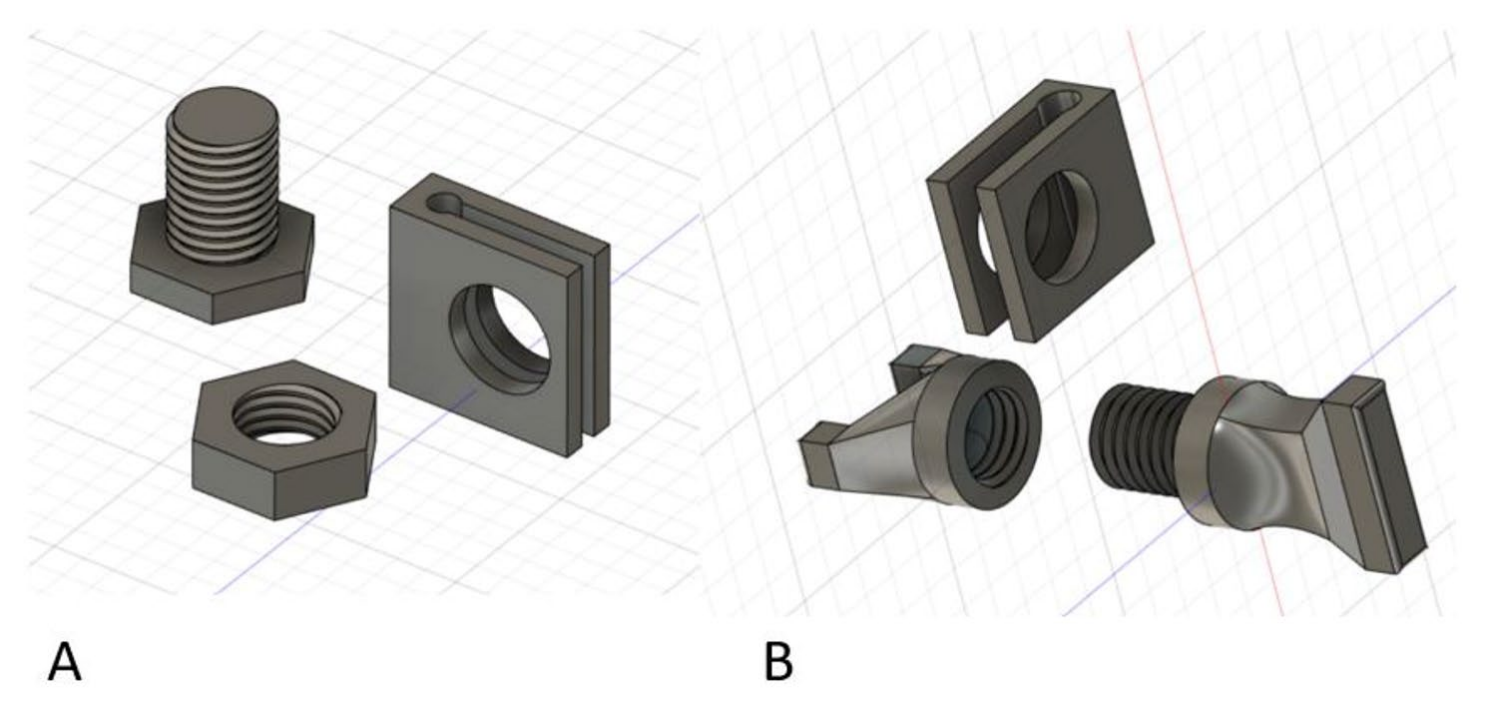
Based on the findings of Fig. 5, this Fig. 6A image demonstrates the M16 screw, nut, and pin clamp to prevent migration up or down on the metal pin. The screw threads were offset − 0.1 mm on both sides and the edge had a 0.1 mm fillet to facilitate mechanical function after 3-D printing. Figure 6B demonstrates a modified design that keeps the surgeon’s hands or wrenches away from the extension cylinders or blocks to facilitate application

Figure 5 A demonstrates another attempt at reinforcing the pin-to-block component to allow for modularity while incorporating the extension rod component. This design was limited in how far the cylinders could provide length and would be applicable in a limited number of fractures. Figure 5 A also demonstrates two cross holes where 3-D printed screws would compress the plastic clip component onto the 5.0 mm pin. However, the PLA material could not maintain a strong enough friction hold on the stainless-steel pin to prevent twisting and rotating about the stainless-steel pin with manual loading of the cylinders. Of note, the compression of this clip mechanism did prevent manually pulling the clamp off the pin once a 3-D printed hex-head screw and nut were secured through the clamp cross-hole. This finding led to the development of the components in Fig. 6.

**Fig. 7:**
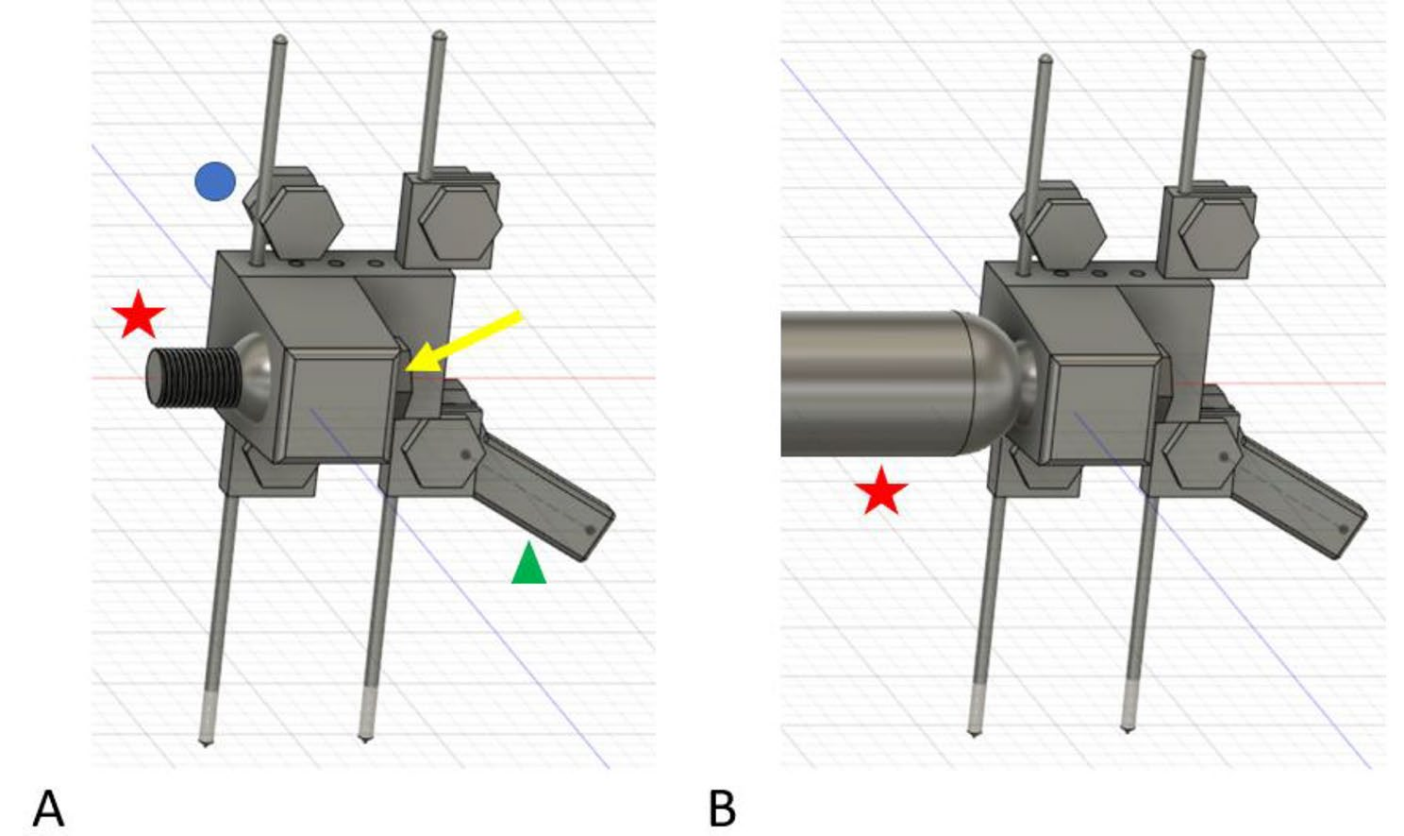
Figure **A** demonstrates the free-floating ball-screw (red star) in the pin-to-block component with the compression fixation hex screw (yellow arrow) fully tightened maintaining that press-fit angle of the ball-screw. The blue dot represents where a clamp would be applied between the hex-head screw and nut from Fig. 6 to prevent upward and downward migration on the metal pins. Two wrenches (green triangle) could tighten the screw-nut to secure the clamp on the pin. Figure **B** demonstrates an extension rod cylinder (red star) screwed into the ball-screw component to create length with the external fixator

Returning to the idea of an articulating ball joint, Fig. 7 demonstrates a solid ball with screw shaft extension that becomes compressed into the pin-to-block component. Figure 7A demonstrates a reinforced pin block secured with multiple Fig. 6 clips above and below the pin-to-block component. A free-floating ball with attached threaded shaft provides greater modularity when an extension rod cylinder is screwed into the thread component on the ball (Fig. 7B). The free-floating ball was compressed in the desired orientation with a large compression threaded screw fixating to the block mechanism and compressing the sphere into the desired orientation (yellow arrow in Fig. 7A). This was an improvement in modularity fixation while resisting slippage, however, the threaded ball mechanism was off axis to the pins similar to the design in Figs. 4D and 5. Although improved, this design was still limited with settling and slippage with loading even after making the sphere a roughened surface to improve friction fit with the compression screw. This may be acceptable for a Delta frame around an ankle fracture or angled external fixator construct, but this would create a bending and torsional moment about a linear external fixator design which would incorrectly reduce a long bone fracture. Again, the modularity aspect created a weak point in the design that allowed for shifting and settling of the components with loading which would not be acceptable for fracture reduction. Similarly, the modularity designs were more complex for 3-D printing manufacturing.

**Fig. 8:**
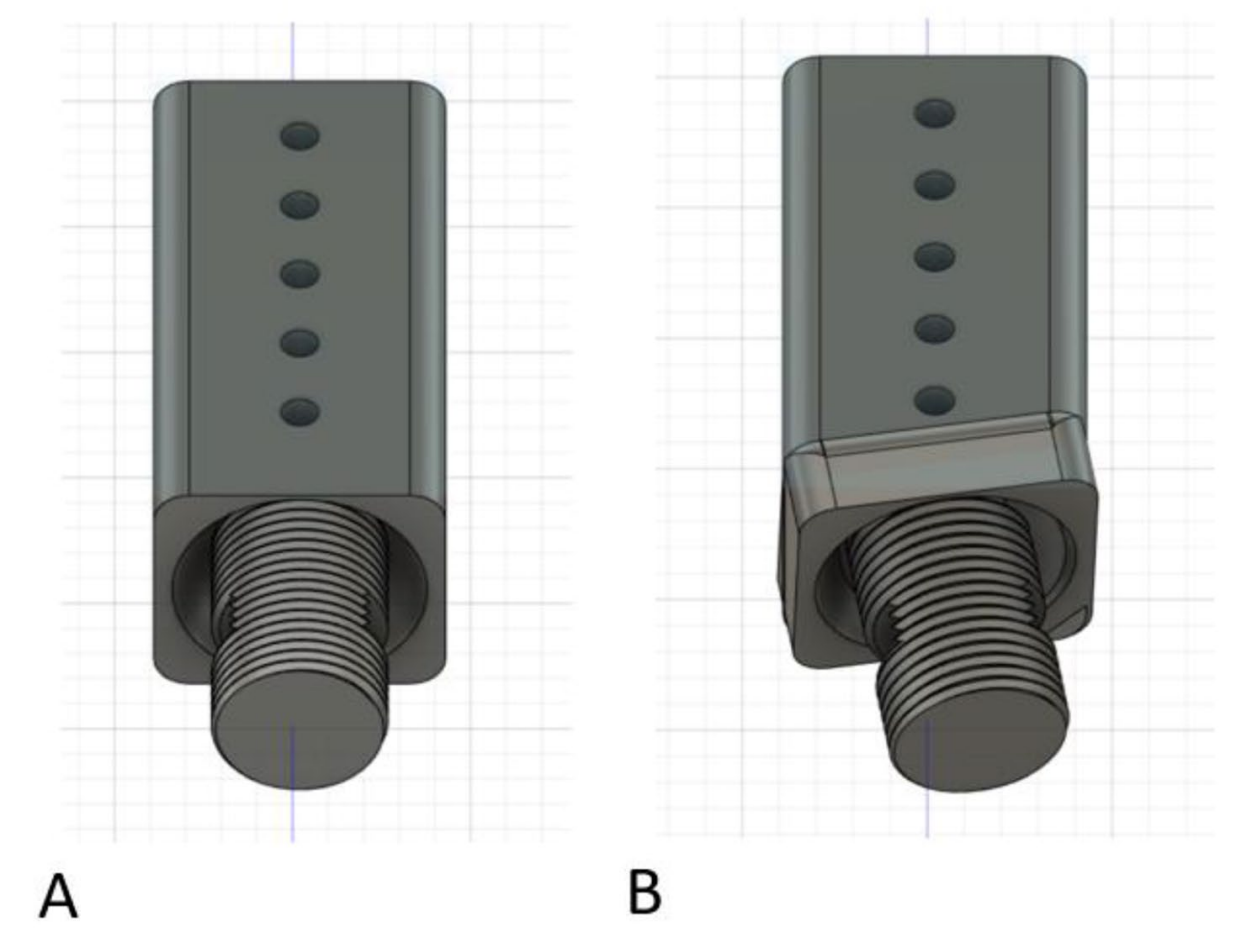
Final pin-to-block design that incorporated fixed angle pin-to-block constructs. Figure **A** demonstrates the orientation for a linear external fixator. Five holes in the block provided for many different metal pin arrangements during surgery. Figure **B** demonstrates an example of an angled block with 20-degree lateral angulation and 20-degree inferior angulation. This could be used for a Delta frame external fixator construct around an ankle. The opening in the threaded component accommodates a de-rotation wedge

It became apparent at this point in the design process that a pin-to-block component needed to have two fixed configurations (Fig. 8). One for linear external fixator constructs in-line with the stainless-steel pins and one at various separate angles to accommodate angled external fixator designs such as an ankle Delta frame. The surgeon would select the desired pin-to-block component (straight versus angled) based on the desired fracture characteristics and location. The PLA material was insufficient to support manual loading when trying to create an all-in-one modular design that would allow for linear and angled constructs with the presented designs. By utilizing a fixed-angle separate block design, based on the desired fracture to be treated (linear versus angled), the interface with the extension rod component could be much stronger, co-axial, fixed-angle, and better resist applied forces. Similarly, the extension rod components needed sizes at least two times that of a 10 mm carbon fiber rod in diameter to replicate stiffness with manual loading. Similarly, setting print infill to 100% improved loading stiffness. These modifications compensated for the weaker PLA material properties with an improved design for simplified 3-D printing.

**Fig. 9:**
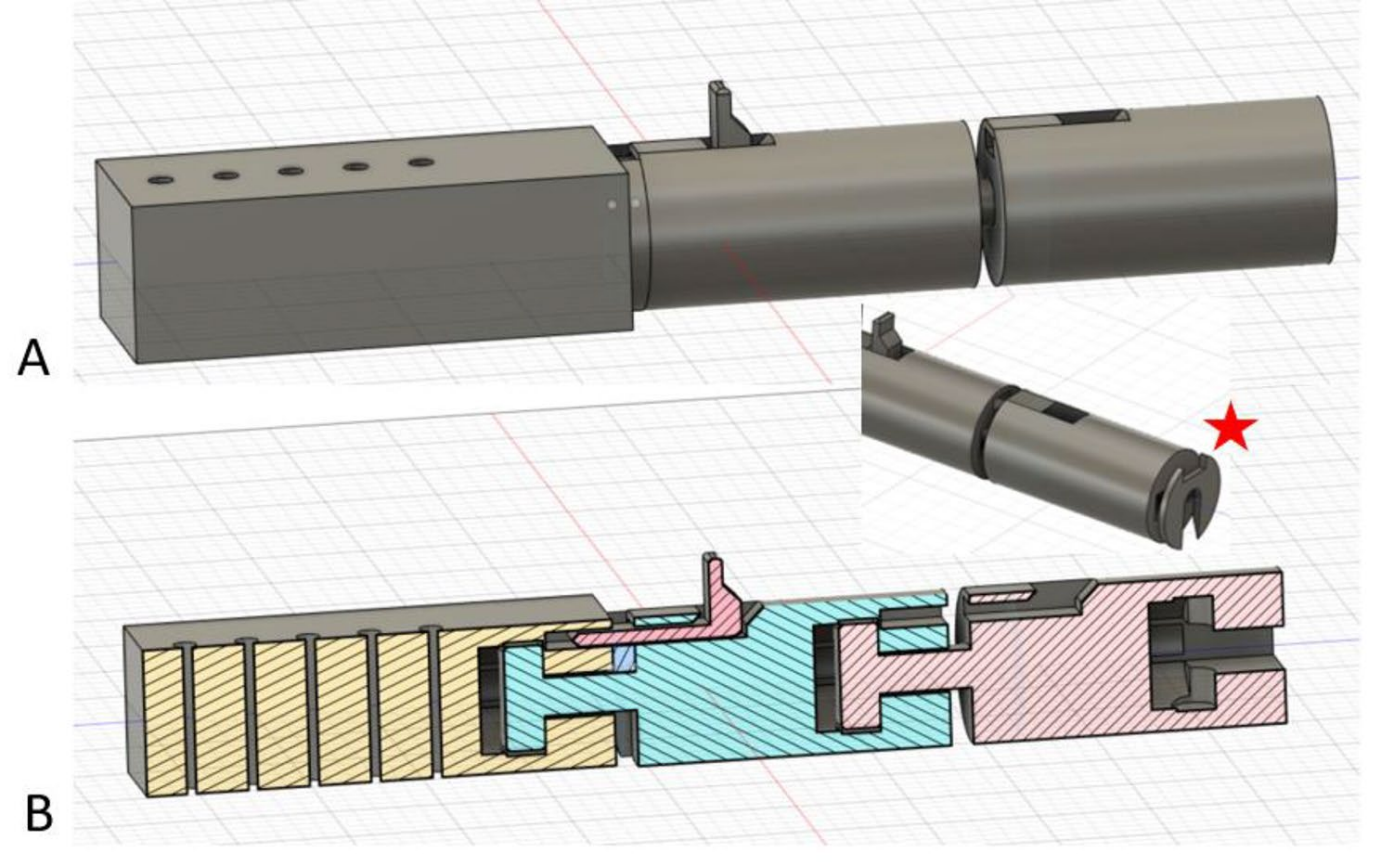
Figure A demonstrates a version of the fixed pin-to-block design interfacing with interlocking blocks to create an extension rod of expandable length. The Figure **A** inset image demonstrates how a wedge (red star) would be hammered between the interlocking components to make a secure fit. The flat broad pin on top of the components would be hammered in place to maintain the wedge fit and prevent torque forces

Attention then began to focus on the rod extension element of the external fixator. Figure 5 previously demonstrated two telescoping cylinders. This design only allowed for two cylinders, and they were restricted in length by the print bed height. This combined length was insufficient for lower leg fractures and did not accommodate torsional forces. Designs then began to focus on interlocking components that could be combined to build-out any length of external fixation rod. Figure 9 illustrates an interlocking design created by twisting components into each other to create length once they were locked into position. Circular wedges would be hammered between the interlocking components to provide rigidity and maintain length of the construct. These wedges would be secured in place and prevent torque moments via a flat broad pin that would be hammered into place over the top of the circular wedge. This broad pin would span the wedge and two interlocking bodies to maintain positioning and prevent torque forces. However, to allow the components to interlock with a tight fit meant that the wedge could only be 5 mm in width at its maximum width-size given the length limitations of the interlocking block design. To accommodate the interlocking mechanisms in the extension component, the extension components were too long to allow for fine fracture reductions which can involve millimeter changes in length. Therefore, this design was also excluded.

Design iterations began to explore a telescoping cylinder component that allowed for extension with a broad range of millimeter reduction lengths. Similarly, the threaded interlocking cylinders of Fig. 4D were revisited to provide more stability compared to the twisting interlocking components of Fig. 9. The cylinders would also allow for a spherical end cylinder to interface into a cup in the fixed pin-to-block component improving contact area and interface strength at this design weak point. Although it worked well for the Fig. 6B pin clamps to control height on the pin, the 3-D printed PLA components would not allow for tightening a mechanism to maintain extension rod length with various cylinders. However, PLA performed very well as a ridged cross-wedge maintaining rod length of telescoping cylinders to provide various lengths.

### Final Design

**Fig. 10:**
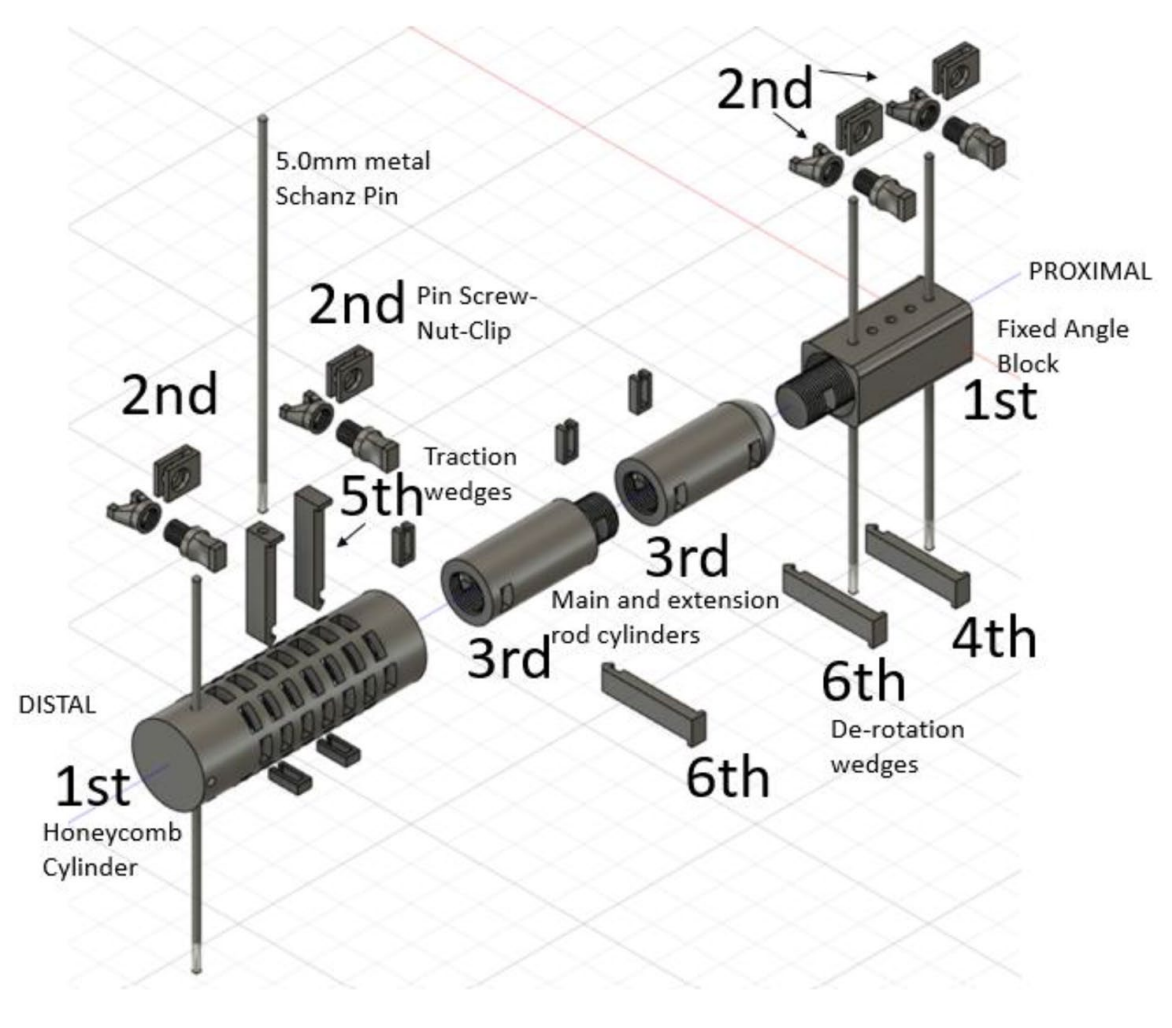
An exploded diagram of the final external fixator design that met the study objectives for fracture stabilization. After 5.0 mm metal pins are placed in stable bone, the first step is to place the fixed angle block and honeycomb cylinder at the desired height on the pins. These components can then be secured in place with pin screw-nut-clips. The main cylinder with or without extension elements would begin deep within the honeycomb cylinder. The extension cylinder would then be threaded and secured with a de-rotation wedge into the fixed block. Traction is applied and traction wedges are placed distal to the extension cylinder to maintain length. An additional metal pin can be placed through a unique wedge, with a hole through it, in the distal aspect of the honeycomb to facilitate final traction and fracture reduction. Finally, any remaining de-rotation wedges, traction wedges, and cross-clips are applied

Figure 10 demonstrates the final design that provided the ability to span a fracture area while maintaining fracture length, preventing bending forces, and resisting torque while accommodating for the limitations of 3-D printing with a FDM desktop printer and PLA filament. Further, this device allows for angled modularity to address many different fracture patterns on the upper and lower extremities. Similarly, the device interfaces with 5.0 mm partially threaded metal pins.

There are multiple unique features to this novel design. A fixed pin-to-bar component is secured on metal pins with or without Fig. 6 clips. In the prototyping process, it was discovered that the imperfections of 5.1 mm holes made in the block for a 5.0 mm metal pin had excellent press fit. The assembly needed to be gently hammered to the desired location on the pins. The fit was so secure, application of the Fig. 6 clips was optional for manual loading. Multiple fixed block holes allow for different metal pin configurations. The block had a threaded screw with a window attached at a fixed angle (Fig. 10). A conforming cylinder is secured into the fixed block conforming to a cup in the receiving block to improve fit, stability, and material contact area. The window accommodates a de-rotation wedge to make the connecting components one element.

The desired external fixator length is built out with the desired size and number of extension cylinders. Cross-wedges with securing clips are hammered through the windows to prevent rotation and torque forces of the cylinders in the final step of external fixator placement. These de-rotation wedges and traction wedges measure 2-7 mm in thickness to allow for a wide range of reduction lengths through the cylinder windows.

The Honeycomb cylinder has several important features. The cylinder has several 5.2 mm holes on the distal end to facilitate different angulations and orientations (0^O^, 10^O^, 20^O^, and 30^O^) for a single 5.0 mm pin. The honeycomb cylinder surrounds the distal aspect of the main or extension rod cylinder construct. The overlapping and advancing rectangular windows allow for numerous wedge placements in increments down to 2 mm to facilitate a wide range of fracture reduction lengths with fine length adjustments. The wedges were originally designed with rectangular cutouts to accommodate cross-clips to hold their position through the cylinder. A hairpin or screw nut mechanism could also be utilized to secure these wedges, however, with load applied, a compression fit was obtained that also assisted in holding the wedges securely in position.

### Method of Application

1. The fixed angle block (either straight or angled based on the fracture being treated) and honeycomb cylinder are placed at the desired height on the metal pins with or without clips and screw-nut combinations to maintain construct height on the metal pins (Fig. 10).
2. The extension rod cylinders are screwed together and sized deep within the honeycomb cylinder at its distal base based on the estimated length needed for fracture reduction. The honeycomb can then be rotated into alignment with the fixed block.
3. The main cylinder (with two de-rotation windows and a convex end) is always utilized in this external fixator construct, however, the extension rod cylinders (single de-rotation window) are optional based on the length of the fracture to be reduced.
4. The main cylinder is retrieved out of the honeycomb cylinder, fully screwed into the fixed proximal block, and a 7 mm thick wedge is applied with hammer or mallet to fix this construct and prevent rotation. A cross-clip is applied to hold the wedge in position.
5. At this point, traction is applied to reduce the fracture. Of note, the extension rod cylinder needs to start maximally deployed into the base of the honeycomb before traction is applied to allow for the maximum overlapping distance of the honeycomb cylinder and extension rod cylinders.
6. While one surgeon holds the construct at length, and thereby pulls the fracture to length, another surgeon hammers in wedges through the honeycomb cylinder windows to maintain that length just distal to the extension rod assembly. In this manner, when manual traction is released, the extension rod rests on the wedge passing through the window in the honeycomb component. The subsequent distal windows can be filled in the honeycomb cylinder to better distribute forces with load.
7. During this process, once the extension cylinder has been withdrawn slightly from the base of the honeycomb, a unique 7 mm wedge with a 5.2 mm hole going through the wedge can be applied through a distal rectangular window to allow for a second metal pin fixation in bone in the distal fragment. This will improve construct strength and can be placed in many different orientations and locations based on the honeycomb windows.
8. The process is repeated pulling traction and hammering in wedges of progressively smaller thickness until the desired traction is applied and fracture reduction obtained.
9. Finally, the last wedges are placed through all the de-rotation windows in the extension cylinders to lock the orientation of the 3-D printed external fixator and prevent components from unscrewing.
10. Cross-clips are applied to all the wedges to secure wedge position. This concludes application of this completely 3-D printed external fixator.

## Discussion

We have described the development of a completely 3-D printed external fixator device. The device meets the orthopaedic goals of maintaining length, alignment, and rotation of a fracture. The traction wedges are placed to maintain fracture length, the cylinder construct functions to prevent bending forces, and the de-rotation wedges are secured in place to prevent torque. The 3-D printed nature of this design and concept allows for great modularity and application to a diverse range of potential clinical scenarios. Further testing is needed for mechanical and clinical assessment. However, the final presented designed product could account for provisional/temporary fixation and definitive fixation purposes in mechanical and clinical testing.

**Fig. 11:**
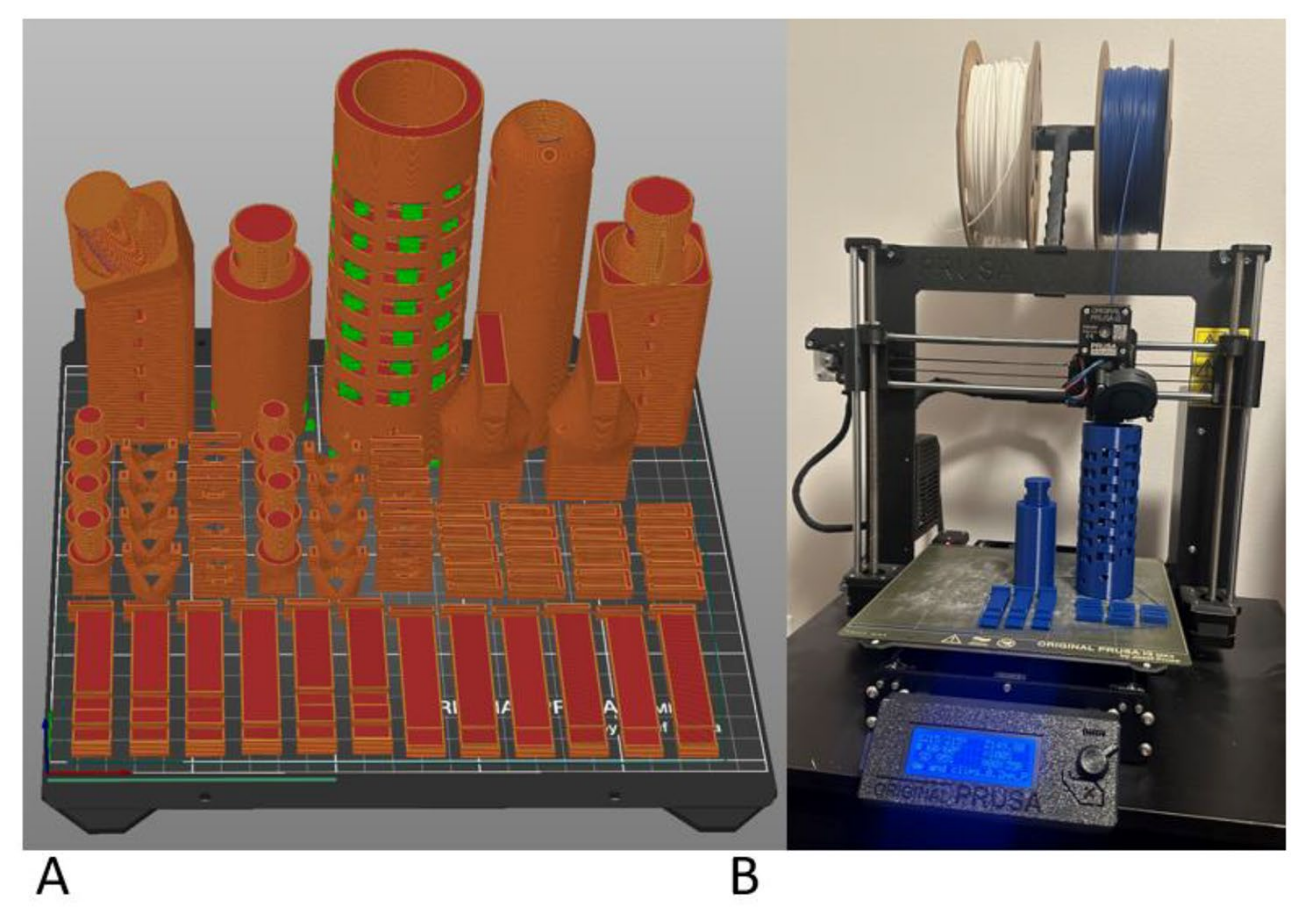
Figure **A** demonstrates an example of a final print bed layout in PrusaSlicer 2.5.1. All components fit on a single print bed even when including an additional angled fixed block, an extension rod, and multiple screws which would not be necessary for many fracture patterns. The green areas in the windows of the cylinders and thread windows are the only scaffolding that needs to be removed in post-processing. Figure **B** demonstrates that scaffolding in the honeycomb cylinder was painted in each window to facilitate hemostat or clamp access for easy removal

The final device was developed over 1-year of design, prototyping, and review. The final presented design allows for significant customization in what the user may need to print to treat a diverse range of fractures and injuries. A common external fixator kit capable of treating many common fracture types of the lower and upper extremities prints in less than two days and needs only one print bed for printing all necessary supplies (Fig. 11). Minimal post-processing is required with this design and only involves using a hemostat or clamp to remove window supports in the printed components.

**Fig. 12:**
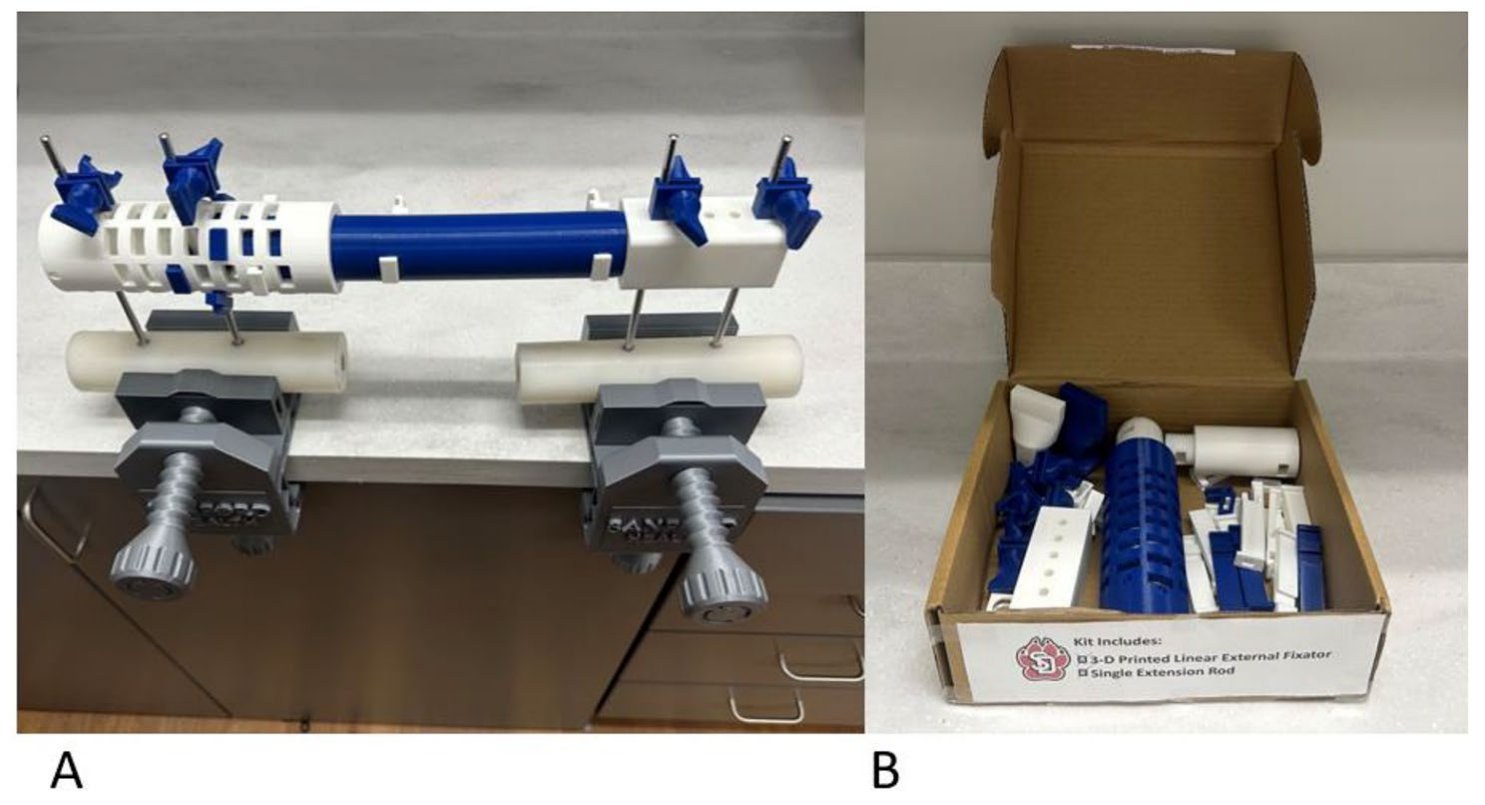
Figure 12A demonstrates an entire 3-D printed external fixator. The fixed block and honeycomb cylinder are white. The main cylinder and extension rod are blue. De-rotations wedges are white and traction wedges are blue. The device is fixed to metal Schanz pins secured in polyurethane dowels to mimic bone. Figure 12B demonstrates how a different deconstructed kit could fit within a standard PLA filament box to facilitate shipping and storage

This example external fixator kit at 100% infill uses approximately 550 g of PLA filament and has a cost of approximately $10.24 in filament material (Fig. 12A). The device is composed of PLA and therefore is radiolucent on X-rays and safe around magnetic resonance imaging systems. The presented example made of PLA is also a much lower weight compared to a similar medical grade external fixator. This could potentially facilitate patient mobility, transfers, and medical staff care with a 3-D printed external fixator. The dis-assembled items can be easily stored in a filament spool box facilitating shipping and storage of printed components (Fig. 12B). Finally, the only additional operative tool needed for this external fixator application is a mallet or hammer, which is a commonly used item in orthopaedic operating theaters.

The ability to 3-D print a low-cost external fixator could be a disruptive technology for multiple different aspects of orthopedic fracture care. During natural disasters such as earthquakes, hurricanes, and tornadoes, medical care facilities can become overwhelmed with the number of patients with fracture care needs [5]. This problem is exacerbated in countries that lack sufficient access to advanced orthopedic care. Low-income or developing countries are another target area that could benefit from a low cost 3-D printed external fixator [8, 16]. During times of political unrest or global conflict, medical facilities are in need of advanced orthopedic equipment [16]. Forward operating military units are also commonly functioning with limited medical supplies. They could, in theory benefit from a print on-demand system that did not require transporting or storing significant amounts of potentially unused medical equipment [17]. Similarly, NASA has already incorporated 3-D printers on the International Space Station to facilitate creating needed equipment on-demand [7]. During long-duration space flights, it would be impractical to transport an entire orthopedic surgical theater and the associated equipment that may never be utilized. As various space exploration entities push further away from Earth, it will also be difficult to have a diverse array of medical specialists on every flight. Future space exploration could benefit from a print on-demand external fixation device. The external fixator is a relatively simple medical device to apply for a wide range of fracture patterns.

Medical devices go through rigorous regulatory and review processes before approval in different countries to ensure safety. In the United States, when 3-D printing is performed by industry and sold to providers, the parts are medical devices regulated by the US Food and Drug Administration (FDA) [18]. When they are 3D printed in a Health Care Facility, at present there is no official guidance from the FDA. However, the FDA has expressed interest in providing guidance [19]. Thus, 3-D printing presents a new challenge for medical professionals with on-demand delivery of medical devices [12]. Caution should be taken by anyone adopting these technologies for medical care, and a quality system should be implemented. For example, having the files printed on the same types of FDM printers along with verified filament types would improve the standardization process if multiple locations were printing these devices. Another safety concern with any medical device is infection risk. Previous studies have demonstrated that PLA components from desktop printers can be sterile at the time of printing due to the heat from the extruder in fused deposition modeling [20]. A benefit of the proposed medical device in this study is that the entire device is external to the body in an already non-sterile area.

Although this device has many benefits, there are several limitations in the current design that will need improvement in future modifications and enhancements. The current design requires a strong understanding of the various component parts of the kit and the sequence of application to obtain a successful fracture reduction outcome. Multiple wedges are needed to secure the components at length and prevent rotation. A hammer or mallet would be a necessary component for application, but this item is commonly found in operating rooms. Similarly, a hemostat or clamp are commonly used in operating rooms and would be needed to remove the window scaffolding. Having modularity in a single print design for various angles would be ideal, but this was not able to be accomplished in the current study. The compact deconstructed size of the kit facilitates shipping and storage. Similarly, the extension cylinder can be stored within the honeycomb component to improve packing. However, these are still considerations and limitations when trying to deliver these items across great distances. Alternatively, a 3-D FDM printer on location could print on-demand but these locations still need access to supplies of PLA filament. Similarly, a medical team making these external fixators on-site would need to have a basic understanding of 3-D printing skills and processes.

## Conclusion

This manuscript details the process of developing and creating a novel completely 3-D printed external fixation device designed to meet the requirements for orthopedic fracture stabilization. Future studies will be needed on the mechanical properties of this device. Furthermore, clinical studies and radiology studies assessing fracture reduction would also be needed before wide-scale use. This orthopedic idea, initiative, and application have significant scalability and disruptive potential based on its utilization of low-cost desktop 3-D printing technology and availability to a global 3-D printing community. Devices, like this proposed design, could benefit a diverse and wide range of patients in situations involving high fracture volume and remote care applications where medical grade external fixator supplies may be limited or unavailable. As the technology and ideas in this orthopaedic area continue to evolve and improve, so will the fracture care in these underserved and remote locations.

## Data Availability

Datasets are digital and may be accessed at any time by contacting the corresponding author.

## Abbreviations

3-D: Three Dimensional
FDM: Fused Deposition Modeling
PLA: PolyLactic Acid
